# Insights on SARS-CoV-2 Molecular Interactions With the Renin-Angiotensin System

**DOI:** 10.3389/fcell.2020.559841

**Published:** 2020-09-16

**Authors:** Larissa Braga Costa, Lucas Giandoni Perez, Vitória Andrade Palmeira, Thiago Macedo e Cordeiro, Victor Teatini Ribeiro, Katharina Lanza, Ana Cristina Simões e Silva

**Affiliations:** Department of Pediatrics, Interdisciplinary Laboratory of Medical Investigation, Faculty of Medicine, Federal University of Minas Gerais (UFMG), Belo Horizonte, Brazil

**Keywords:** Renin Angiotensin System, SARS-CoV-2, ACE2, COVID-19, Ang II, Ang-(1-7), AT1 receptor, pathogenesis

## Abstract

The emergence of SARS-CoV-2/human/Wuhan/X1/2019, a virus belonging to the species *Severe acute respiratory syndrome-related coronavirus*, and the recognition of Coronavirus Disease 2019 (COVID-19) as a pandemic have highly increased the scientific research regarding the pathogenesis of COVID-19. The Renin Angiotensin System (RAS) seems to be involved in COVID-19 natural course, since studies suggest the membrane-bound Angiotensin-converting enzyme 2 (ACE2) works as SARS-CoV-2 cellular receptor. Besides the efforts of the scientific community to understand the virus’ molecular interactions with human cells, few studies summarize what has been so far discovered about SARS-CoV-2 signaling mechanisms and its interactions with RAS molecules. This review aims to discuss possible SARS-CoV-2 intracellular signaling pathways, cell entry mechanism and the possible consequences of the interaction with RAS components, including Angiotensin II (Ang II), Angiotensin-(1-7) [Ang-(1-7)], Angiotensin-converting enzyme (ACE), ACE2, Angiotensin II receptor type-1 (AT1), and Mas Receptor. We also discuss ongoing clinical trials and treatment based on RAS cascade intervention. Data were obtained independently by the two authors who carried out a search in the PubMed, Embase, LILACS, Cochrane, Scopus, SciELO and the National Institute of Health databases using Medical Subject Heading terms as “SARS-CoV-2,” “COVID-19,” “Renin Angiotensin System,” “ACE2,” “Angiotensin II,” “Angiotensin-(1-7),” and “AT1 receptor.” Similarly to other members of *Coronaviridae* family, the molecular interactions between the pathogen and the membrane-bound ACE2 are based on the cleavage of the spike glycoprotein (S) in two subunits. Following the binding of the S1 receptor-binding domain (RBD) to ACE2, transmembrane protease/serine subfamily 2 (TMPRSS2) cleaves the S2 domain to facilitate membrane fusion. It is very likely that SARS-CoV-2 cell entry results in downregulation of membrane-bound ACE2, an enzyme that converts Ang II into Ang-(1-7). This mechanism can result in lung injury and vasoconstriction. In addition, Ang II activates pro-inflammatory cascades when binding to the AT1 Receptor. On the other hand, Ang-(1-7) promotes anti-inflammatory effects through its interactions with the Mas Receptor. These molecules might be possible therapeutic targets for treating COVID-19. Thus, the understanding of SARS-CoV-2 intracellular pathways and interactions with the RAS may clarify COVID-19 physiopathology and open perspectives for new treatments and strategies.

## Introduction

SARS-CoV-2/human/Wuhan/X1/2019 was firstly described in December 2019, in Wuhan, China (Zhou et al., 2020). The World Health Organization (WHO) declared the Coronavirus Disease 2019 (COVID-19) pandemic on 11 March 2020. The scientific community is concentrating efforts to better understand COVID-19 natural course, as well as its pathogenesis and possible therapeutic strategies. SARS-CoV-2 is the etiological agent of COVID-19 and belongs to the *Severe Acute Respiratory Syndrome-related coronavirus* species (Coronaviridae Study Group of the International Committee on Taxonomy of Viruses, 2020), which are RNA-enveloped viruses from the *Coronaviridae* family.

Besides SARS-CoV-2, only SARS-CoV and MERS-CoV outbreaks, in 2003 and in 2012 have been described to cause the life threatening diseases, the Severe Acute Respiratory Syndrome (SARS) and the Middle East Respiratory Syndrome (MERS), respectively. From 1965, when the first coronavirus was identified in patients with common cold (Tyrrell and Bynoe, 1965) until now, seven coronaviruses are described to cause human diseases: HCoV-OC43, HKU1, HCoV-229E, HCoV-NL63, SARS-CoV, MERS-CoV, and SARS-CoV-2 (Wang Q. et al., 2020). These pathogens are zoonotic viruses that jumped species boundaries (Zhang and Holmes, 2020), leading to human diseases. The coronaviruses share the potential to outbreak as pandemics, but small and crucial genetic mutations directly influence their infectivity. On this wise, viruses are obligate intracellular pathogens and their survival relies entirely on host cell machinery control to synthesize and organize their structural components. Viral infections are complex processes, which initiate with viral recognition and attachment to the host cell receptor. SARS-CoV and SARS-CoV-2 have several genetic similarities (Lu et al., 2020; Zhang and Holmes, 2020) and both attach to Angiotensin-converting Enzyme 2 (ACE2), which is anchored to plasma membrane via its transmembrane domain (Hoffmann et al., 2020; Wan et al., 2020). SARS-CoV-2 envelope is composed of two proteins to structure maintenance (membrane and envelope proteins), and the spike glycoprotein (S), which mediates host cell entry. SARS-CoV-2 entry mechanisms are still under investigation and further characterization is needed to best describe SARS-CoV-2 hypothetical mechanisms. In this review, we present published studies about SARS-CoV-2, SARS-CoV, ACE2 and other mediator components of the first step of infection. Furthermore, we also show how the binding of SARS-CoV-2 may trigger a Renin Angiotensin System (RAS) imbalance due to its binding to ACE2, possibly contributing to the pathogenesis of COVID-19.

Angiotensin-converting Enzyme 2 is an important component of the RAS (Donoghue et al., 2000; Tipnis et al., 2000) a molecular system composed of a wide range of peptides, enzymes, and receptors (Simões e Silva and Flynn, 2012). Angiotensin II (Ang II) and Angiotensin-(1-7) [Ang-(1-7)] are the major effector molecules of the two main RAS pathways: the classical axis, composed by the Angiotensin-converting enzyme (ACE), Ang II and Angiotensin II receptor type 1 (AT1), and the alternative axis, which includes ACE2, Ang-(1-7), and the Mas receptor (MasR) (Santos et al., 2003). Under physiological circumstances, the homeostatic state is achieved due to the counter-regulatory actions of these two arms. Although there’s hardly any published evidence in this regard, SARS-CoV-2 binding to ACE2 might result in ACE2 availability reduction, leading to ACE2/Ang-(1-7)/MasR axis downregulation and consequent exacerbation of the ACE/Ang II/AT1R axis (Rodrigues Prestes et al., 2017; Lanza et al., 2020). This may cause important pulmonary, immune and hematological disturbances. Thus, RAS imbalance might not only be a consequence of the disease, but a crucial step of COVID-19 pathogenesis (Lanza et al., 2020).

This counter-regulatory pattern might explain both symptomatology and epidemiological patterns of risk groups. Usually after a 5-day incubation period, the most common observed symptoms are fever, dry cough, tiredness, and neurological manifestations, including anosmia, ageusia and dysgeusia (Lechien et al., 2020; Li Q. et al., 2020). Other signs and symptoms can also be found, including sputum production, headache, hemoptysis, diarrhea, dyspnea, lymphopenia and important changes in lung imaging investigation (Rothan and Byrareddy, 2020). Evidence shows that the symptoms related to severe pneumonia are mainly due to an exaggerated immune response and cytokine storm (Mehta et al., 2020). These findings are closely related to pulmonary tissue damage, inflammatory response and hematological disturbances. The three steps pathophysiology proposed in this article link these phenomena with the RAS imbalance hypothesis (Lanza et al., 2020).

## Methods

The references were obtained independently by the two authors, who carried out a comprehensive and non-systematic search in the PubMed, Embase, LILACS, Cochrane, Scopus and SciELO databases. Search strategies included Medical Subject Heading terms as: “SARS-CoV-2,” “COVID-19,” “Renin Angiotensin System,” “ACE2,” “Angiotensin II,” “Angiotensin-(1-7),” and “AT1 receptor.” The search emphasized recent articles, published case series, consensus statements, guidelines, meta-analyses, systematic reviews and prospective cohort studies, critically reviewed and selected by the authors.

## Signaling and Cell Entry Mechanisms of SARS-CoV-2

### Summarizing Current Knowledge About How SARS-CoV-2 Enters Host Cell

Several studies reported membrane-bound ACE2 as SARS-CoV-2 receptor (Hoffmann et al., 2020; Liu et al., 2020; Zhou et al., 2020). The binding of SARS-CoV-2 to its functional receptor, the membrane-bound ACE2, facilitates the virus entry into the cell. Viral binding to ACE2 involves distinct domains of the spike (S) protein. Due to a multi-step variation of its conformational state, SARS-CoV-2 is able to attach itself to the cell surface, firmly binding to ACE2 and starting the membrane fusion step (Letko et al., 2020). Other membrane proteins are essential to viral entry into the cell through priming and activating of the S protein. Firstly, SARS-CoV-2 has a FURIN cleavage site, which is absent in SARS-CoV (Coutard et al., 2020). This site may enhance binding affinity between the SARS-CoV-2 S protein and the human membrane-bound ACE2. Moreover, SARS-CoV-2 receptor-binding domain (RBD) has significant differences in its amino acid sequence if compared to SARS-CoV RBD, leading to higher affinity of SARS-CoV-2 to membrane-bound ACE2. The binding of SARS-CoV spike protein occurs with less affinity, due to its naturally less accessible conformation (Shang et al., 2020a). Additionally, SARS-CoV-2 exploits a cellular serine protease, TMPRSS2, and, in a smaller rate, an endosomal cysteine protease, cathepsin B, and L (CatB/L) (Hoffmann et al., 2020). In this sense, the TMPRSS2 downregulation as a cell self-defense mechanism (Guzzi et al., 2020) may be overpassed by SARS-CoV-2 through the CatB/L endosomal pathway (Kawase et al., 2012). Simultaneous treatment *in vitro* with a serine protease inhibitor and a cathepsin inhibitor blocks both cell entry and the multistep growth of SARS-CoV-2 in human airway epithelial cells (Hoffmann et al., 2020).

Although the endocytosis-mediated entry is not a consensus, growing evidence points to mechanisms for this pathway. Endosomal transport through the cell depends on H^+^ - ATPases activity, which are coupled to the endosomal membrane due to the fusion of circulating vesicles carrying these proton pumps (Bright et al., 2016; Naslavsky and Caplan, 2018). Subsequently, endosomes can fuse with lysosomes. In the meantime, internal pH decreases, inducing irreversible conformational changes by a variety of mechanisms, including protonation of histidine residues and salt bridges. Furthermore, endosomal cathepsin L proteolysis might act as a third priming event (Simmons et al., 2005). This buries S fusion peptide (FP) and exposes it to the endosomal membrane (Rachakonda et al., 2007; White et al., 2008). The endosomal membrane is then disrupted, forming a pore through which the viral particles translocate to the cytoplasm.

### SARS-CoV-2 and S Protein Structure

The SARS-CoV-2 is an enveloped, positive-strand RNA *Betacoronavirus* of the Coronaviridae family. Genomic sequence analysis of SARS-CoV-2 suggests that the 30 kb genome encodes as many as 14 open reading frames (ORFs) (Gordon et al., 2020). ORF1a/ORF1ab encodes 16 non-structural proteins (Nsp1-16) that form the replicase and transcriptase complex (RTC). The other 13 encode four structural proteins – Spike (S), Envelope (E), Membrane (M), and Nucleocapsid (N) – and nine putative accessory factors.

There are six open reading frame proteins (ORFs), ORF3a, ORF6, ORF7a, ORF7b, ORF8, and ORF10, and the polyprotein ORF1ab (Srinivasan et al., 2020). A study of evolutionary conservation found that the majority of these proteins has either no modifications or a mutation in the peripheral binding region, in comparison to SARS-CoV (Srinivasan et al., 2020). However, unlike most of these protein cited before, the Spike protein of SARS-CoV-2 has significant changes at amino acid sequence if compared to other human coronaviruses (hCoV), including SARS-CoV (Ou et al., 2020; Shang et al., 2020b; Walls et al., 2020; Wang Q. et al., 2020). S is formed by a S1 subunit, responsible for receptor binding, attached to S2, the subunit responsible for membrane fusion, which comprises three subdomains that loop back on each other (Shang et al., 2020a). This might result in essential differences regarding molecular interactions, which enhance the binding affinity of SARS-CoV-2 to ACE2.

**FIGURE 1:**
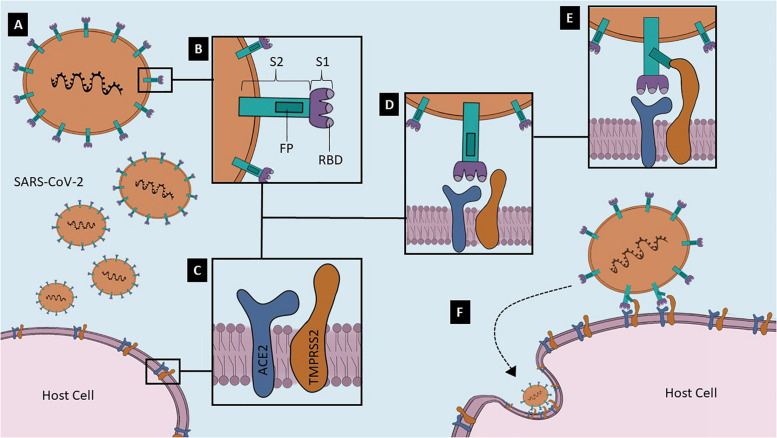
Potential mechanisms of SARS-CoV-2 Spike glycoprotein (S) binding to and invading host cell. **(A)** Virion gets closer to the host cell that expresses a high-affinity binding receptor on its surface. In its native fusion-competent state, the Spike glycoprotein (S) of SARS-CoV-2 is anchored on the virion envelope. **(B)** S is formed by a S1 subunit, which contains the receptor-binding domain (RBD), and by a S2 subunit, comprising the fusion peptide (FP) domain. **(C)** Two of the most important proteins in the host cell surface related to the virus entry are Angiotensin-converting Enzyme 2 (ACE2) and Transmembrane protease/serine subfamily 2 (TMPRSS2); **(D)** Membrane bound-ACE2 is the SARS-CoV-2 receptor, and TMPRSS2 is the S primer. These two host cell proteins probably form a complex on the lipid bilayer; **(E)** S1 RBD attaches to ACE2 and TMPRSS2 cleaves S2 in a step named as priming, leading to the exposure of the FP; **(F)** Viral S protein is anchored not only to virion surface, but also to host plasma membrane. Hence, other S is recruited and the endocytic viral entry process begins. These are representative no-scale images.

Although all viral fusion proteins have a similar conformation at the end of virus-cell membrane fusion, known as trimer of hairpins, they are divided into three classes that differ in structure (White et al., 2008). Coronaviruses S proteins are class I viral fusion proteins, which mean that these proteins are assembled into trimers in their pre- and post-fusion states (White et al., 2008). The trimer of hairpins form a structure called 6HB, a six-helix bundle that approximates target cell and virion membranes (White et al., 2008). Viral S proteins have two functional subunits: S1, the distal receptor-binding subunit, and S2, the fusion machinery subunit (Millet and Whittaker, 2015). In addition, S1 has the RBD and N-linked glycans, which may function as glycan shields, protecting SARS-CoV-2 from antibody recognition (Walls et al., 2020). S1 is the less conserved subunit, reflecting a high selective pressure promoted by the host immune system. SARS-CoV and SARS-CoV-2 S share about 79.6% of amino acid residue sequence in regard to the entire protein, but only 74% comparing the RBD (Ou et al., 2020). On the other hand, S2 has a high sequence identity with other hCoV in some important regions: the short cytosolic tail; palmitoylated cysteines-transmembrane domain; two heptad repeats (HR1 and HR2); and a fusion peptide, a hydrophobic amino acid residue sequence that engages target membrane (White et al., 2008; Millet and Whittaker, 2015; Walls et al., 2020; Figure 1). S1/S2 and S2′ are the two cleavage sites in SARS-CoV-2 S, allowing the activation and priming steps (Hoffmann et al., 2020) through protease actions. S1/S2 also has a four amino acid residue furin-like cleavage sequence (PRRA), indicating that furin, a calcium-dependent serine peptidase, can cleave this site (White et al., 2008; Walls et al., 2020). S2′ contains a residue sequence located upstream the fusion peptide and can be cleaved by a diversity of proteases (Hoffmann et al., 2020; Walls et al., 2020). Although few evidence show the role of these proteolytic activation sites on SARS-CoV-2 life cycle, it is believed that S2′ is cleaved during biosynthesis and S1/S2 is cleaved for the virus to enter the cell (Walls et al., 2020).

### S Protein Binding to ACE2

Angiotensin-converting enzyme 2 is a zinc membrane-bound metalloproteinase that acts as a carboxypeptidase able to hydrolyze Ang I to Ang-(1-9) and Ang II to Ang-(1-7). ACE converts Ang I into Ang II, while ACE2 forms Ang-(1-9) from Ang I. ACE2 differs from ACE regarding its insensitivity to conventional ACE inhibitors (Donoghue et al., 2000; Tipnis et al., 2000; Rodrigues Prestes et al., 2017). Both SARS-CoV and SARS-CoV-2 have ACE2 as their host cell receptor (Lee et al., 2003; Letko et al., 2020), but SARS-CoV-2 S RBD has a receptor binding motif (RBM) capable to attach ACE2 with higher affinity if compared to SARS-CoV S. Studies showed major structural changes that explain this difference (Glowacka et al., 2010; Shang et al., 2020b; Walls et al., 2020; Wrapp et al., 2020): (1) variable ridge loop with four residue motif (Gly-Gln-Thr-Gly) instead of a three motif, allowing additional main-hydrogen bonding between RBM and ACE2 N-terminal helix; (2) SARS-CoV-2 S RBM better insertion into ACE2 hydrophobic pocket, due to novel interactions because of Leu472; (3) Lys31 and Glu35 from ACE2 binding to Leu455 and Gln493 from SARS-CoV-2, respectively (hotspot 31); (4) unique hydrogen bonding between Lys353 from ACE2 and SARS-CoV-2 RBD main chain (hotspot 353) (Shang et al., 2020b). Hence, the high-affinity SARS-CoV-2/ACE2 interaction could be an explanation for the greater infectivity of this virus when compared to others hCoVs.

Figure 1 illustrates the mechanisms of SARS-CoV-2 binding to membrane-bound ACE2 and subsequent entry into the human cell.

### Other Potential SARS-CoV-2 Receptors

Tissue tropism and host range are determined by a variety of factors. Nonetheless, receptor recognition and attachment are essential steps for a viral infection (Maginnis, 2018). Intracellular pathogens usually attach to more than one host cell surface structure that exerts the function of viral receptor. Carbohydrates, such as sialic acid (SiAc), and proteins, integrins and the membrane-bound ACE2 for instance, are common receptors used by viruses. Studies suggest that infection might follow a series of receptor engaging and detachment, until the pathogen interacts with host cells in a high-affinity event (Maginnis, 2018).

Sialic acid is ubiquitously expressed on the surface of host cells and is capable of mediating cell adhesion and transduction-signaling events. SiAc might be the first host cell-virus interaction in SARS-CoV-2 infection. MERS-CoV binds not only to dipeptidyl peptidase 4 (DPP4), its protein receptor, but also to sialic acids (α2,3-linked especially) (Li et al., 2017). Furthermore, SARS-CoV-2 is the first discovered hCoV that has a specific motif in its S able to bind to integrins receptors, integral membrane proteins arranged as heterodimers with alpha and beta subunits. Integrins mediate a variety of mechanisms, including cell adhesion, signaling events, and cytoskeletal rearrangement. In relation to the SARS-CoV-2, these molecules recognize two specific motifs: RGD (Arg-Gly-Asp) and KGE (Lys-Gly-Glu). Although integrin-binding is essential for a variety of human-viruses (Stewart and Nemerow, 2007), no previously described coronaviruses were capable of making these transmembrane proteins as receptors. Therefore, SARS-CoV-2 is the first coronavirus that has a RGD motif S protein present in the RBD of S1 (residues 403–405) (Sigrist et al., 2020). The implications of this finding, however, are still unknown.

The extracellular matrix metalloproteinase inducer (EMMPRIN), also known as CD147, represents another potential receptor for SARS-CoV-2 (Ulrich and Pillat, 2020). Wang K. et al. (2020) showed that SARS-CoV-2 invaded human host cells via CD147 binding (Wang K. et al., 2020). This protein belongs to an immunoglobulin superfamily enrolled in inflammatory processes and virus host cell entry (Pushkarsky et al., 2001; Chen et al., 2005; Sagkan and Akin-Bali, 2020). Differently from ACE2, CD147 is ubiquitously expressed in epithelium and immune cells (Radzikowska et al., 2020). Interestingly, CD147 is upregulated in patients with obesity and diabetes, which might explain, at least in part, why these comorbidities are considered risk factors for severe COVID-19.

### Proteases and Priming

Proteolytic activation of viral fusion-protein is an essential step for membrane fusion in a variety of viruses. Besides allowing the fusion triggering process named as priming, induced viral fusion-protein conformational changes release sufficient energy to overcome the lipid bilayer fusion energy barrier. On that matter, SARS-CoV-2 receptor is a protease distinct of the S primer, once RBD binds ACE2 distant from its action site (Shang et al., 2020b). Therefore, several proteases are capable of priming viral fusion-proteins. Based on previous knowledge about other hCoVs, researchers discovered that TMPRSS2 is essential for SARS-CoV-2 cell entry (Hoffmann et al., 2020). TMPRSS2 belongs to the type II transmembrane serine proteases family (TTSP) and is found on cell surface or in the secretory pathway (Szabo and Bugge, 2008, 2011). Evidence suggests that this protease forms complexes with ACE2 in plasma membrane microdomains (Shulla et al., 2011; Tarnow et al., 2014), corroborating to the hypothesis that TMPRSS2 and ACE2 operate together in SARS-CoV-2 cell entry. Thus, TMPRSS2 might catalyze the cleavage of S2′ after S binding to the receptor.

Cathepsin L, another protease related to a variety of coronaviruses, seems also to be involved in SARS-CoV-2 molecular mechanisms (Ou et al., 2020). This ubiquitously expressed protein is a cysteine protease activated in low pH. Besides acting as an essential protease for several viruses (Millet and Whittaker, 2015), cathepsin L can be found in lysosomes (Turk et al., 2012). SARS-CoV and MERS-CoV, for instance, used cathepsin L to cell entry (Simmons et al., 2005; Qian et al., 2013). The role of cathepsin L in SARS-CoV-2 cell infection is not well defined. Bosch et al. (2008) reported that cathepsin L cleavage site for SARS-CoV S is T678, 11 residues downstream the trypsin cleavage site.

## Renin-Angiotensin System and COVID-19

### RAS Ubiquitous Role in Homeostasis

The Renin-Angiotensin System was first conceived as centered in the local and systemic actions of Angiotensin II (Ang II). However, last decades’ studies regarding the RAS established a complex and dynamic molecular cascade with two fundamental arms, a classical and an alternative axes, and a range of counter-regulatory actions in different organ systems. Its endocrine (tissue-to-tissue), paracrine (cell-to-cell) and intracrine (intracellular/nuclear) effects (Patel et al., 2017) are crucial for cardiovascular, renal, immune, pulmonary, and neural homeostasis (Nehme et al., 2015). The RAS also plays a pivotal role in several pathophysiological disease models, including pulmonary and renal diseases (Simões e Silva and Flynn, 2012; Magalhaes et al., 2018).

The classical axis, comprising the ACE, its main product Ang II and the angiotensin II type 1 (AT1) receptor, mediates the well described body fluid homeostasis through restoration of blood volume. Several lines of evidence, however, attribute a range of deleterious actions to the classical ACE/Ang II/AT1R axis, including enhancement of inflammation, fibrosis, cellular growth, and migration (Rodrigues Prestes et al., 2017). Additionally, this axis triggers vasoconstriction, cardiac hypertrophy and reactive oxygen species (ROS) production (Ardaillou, 1998; Yamada et al., 1998).

Findings on the ACE2/Ang-(1-7)/MasR arm are substantially new. Santos et al. (1988) described Ang-(1-7) production in dog brainstem dependently and independently of Ang II formation, which suggested an alternative route for generating RAS fragments. In the same year, Schiavone et al. (1988) showed the first biological effect of Ang-(1-7), with the heptapeptide was able to release Vasopressin from pituitary-hypothalamus tissue explants (Schiavone et al., 1988). Further, several actions of Ang-(1-7) were successively reported (Santos et al., 2000). However, until 2000, the preferential route of Ang-(1-7) formation was still lacking. In this regard, in 2000, two independent research groups discovered almost simultaneously the enzyme homolog to ACE, named ACE2, as the main responsible for the conversion of Ang II into Ang-(1-7) (Donoghue et al., 2000; Tipnis et al., 2000). Three years later, Santos et al. (2003) determined the G-protein coupled Mas receptor (MasR) as Ang-(1-7) receptor. The high affinity binding of Ang-(1-7) to the MasR is possible after the cleavage of Ang II by ACE2, subtracting the Phenylalanine amino acid (Santos et al., 2018). Discoveries on ACE2 and the MasR resulted in a new conception of the RAS. Currently, the RAS is defined as a dual arm system formed by two counter-regulatory axes, the classical ACE/Ang II/AT1 axis and the alternative ACE2/Ang-(1-7)/MasR arm (Santos et al., 2005). The alternative arm exerts beneficial effects through counter-regulating the RAS classical axis and its effects includes vasodilation, inhibition of cell growth and ROS production, anti-inflammatory, anti-thrombosis and anti-arrhythmogenic actions (Simões e Silva and Flynn, 2012; Simões e Silva and Teixeira, 2016; Rodrigues Prestes et al., 2017).

**FIGURE 2:**
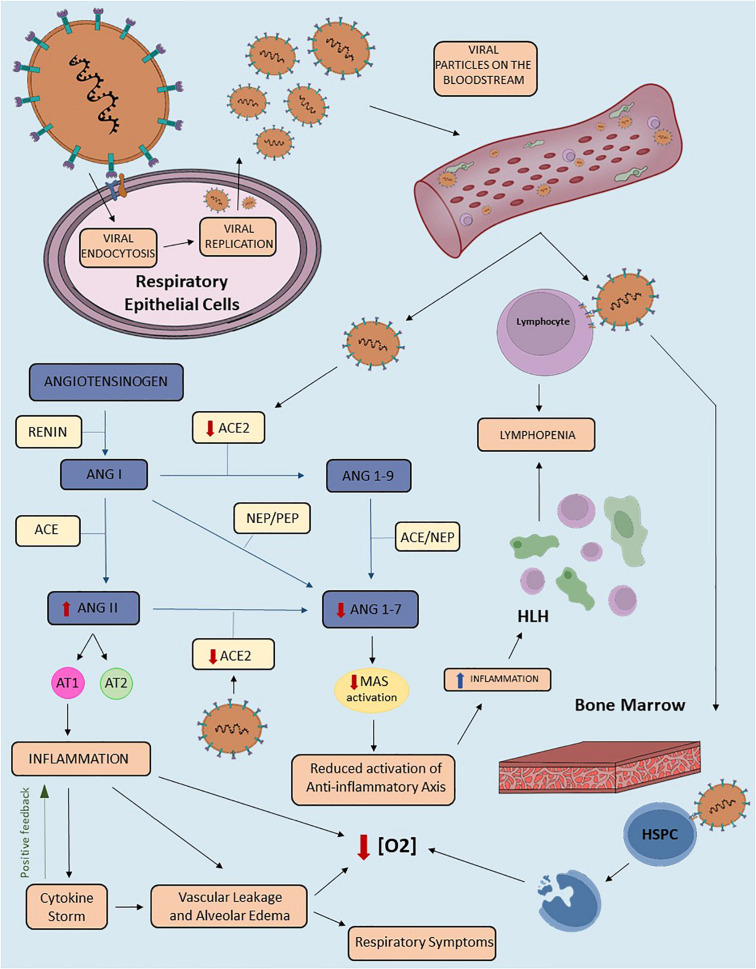
The proposed role of the Renin-Angiotensin System in the pathophysiology of COVID-19. Schematic representation of COVID-19 pathophysiology related to the Renin-Angiotensin System (RAS) imbalance. This figure highlights the downregulation of transmembrane Angiotensin-converting enzyme 2 (ACE2) in SARS-CoV-2 infection. The virus enters the host cell after binding to TMPRSS2 and transmembrane ACE2. Viral replication and release from lung cells to the bloodstream enhance viremia, besides diminishing circulating and transmembrane ACE2 levels. The reduction of ACE2 availability results in RAS imbalance due to downregulation of the alternative axis. Consequently, we have an increase in Angiotensin II (Ang II) and decrease in Angiotensin-(1-7) [Ang-(1-7)] levels. The binding of Ang II to the Angiotensin II type 1 (AT1) receptor triggers inflammatory response, including vascular leakage and alveolar edema, both of which can be amplified by Cytokine Storm Syndrome (CSS). This mechanism may contribute to several clinical presentations of COVID-19, including respiratory signs and symptoms. In addition, the downregulation of the ACE2/Ang-(1-7)/Mas receptor axis reduces the anti-inflammatory effects of the alternative RAS axis. Due to ACE2 expression in mature lymphocytes, SARS-CoV-2 may result in lymphopenia. This finding can also be triggered by hemophagocytic lymphohistiocytosis (HLH) due to intense tissue inflammation. In addition, the invasion of the bone marrow by the virus, specifically of the hematopoietic stem/progenitor cells (HSPC), leads to apoptosis and consequent reduction of oxygen saturation levels. Other mechanisms that may contribute to lower saturation include vascular leakage, alveolar edema and inflammation. ACE2, Angiotensin Converting Enzyme 2; TMPRSS2, Transmembrane protease serine 2; RAS, Renin-Angiotensin-System; ANGII, Angiotensin II; ANG(1-7), Angiotensin (1-7); HLH, Hemophagocytic Lymphohistiocytosis; HSPC, Hematopoietic stem/progenitor cell; [O2], Oxygen concentration; ACE, Angiotensin Converting Enzyme; ANG(1-9), Angiotensin (1-9); ANGI, Angiotensin I; PEP, prolyl-endopeptidase; NEP, neutral-endopeptidase.

Several studies demonstrated the pivotal role of RAS imbalance in disease progression through disruption of the system’s equilibrium (Simões e Silva and Teixeira, 2016). Hence, the reduction of a RAS molecule function or bioavailability might lead to exacerbation of one axis. Indeed, administration of RAS blocker therapy, which inhibits the classical RAS axis, has been also described to enhance the alternative axis in humans and animal models (Simões e Silva and Flynn, 2012). This principle might be an underlying component in many diseases pathogenesis, including COVID-19 (Lanza et al., 2020). Figure 2 shows the potential mechanisms that may link RAS imbalance to signs and symptoms of COVID-19.

### RAS Imbalance and COVID-19 Pathophysiology

Growing evidence supports the role of ACE2 downregulation in COVID-19 pathophysiology and the possible contribution of RAS axes unbalance to COVID-19 natural history (D’Ardes et al., 2020; Gheblawi et al., 2020; Lanza et al., 2020; Verdecchia et al., 2020). It seems that the dynamics of ACE2 and SARS-CoV-2 infection relies on an apparent paradox that depends on the bioavailability of ACE2: either (1) the individual infected by the virus has enough reservoir of ACE2 to resist the depletion of the enzyme and still counteract the deleterious effects of reduced ACE2 levels and activity; or (2) individuals with a small reservoir of ACE2 will not be able to activate the anti-inflammatory axis of the RAS and consequently will suffer from an exacerbated activation of the classical pro-inflammatory arm.

Angiotensin-converting enzyme 2 is significantly downregulated in several experimental studies with induced pulmonary injury (Imai et al., 2005; Kuba et al., 2006; Gu et al., 2016; Sodhi et al., 2019). Its blockade or genetic manipulation resulted in enhanced vascular permeability, neutrophil accumulation, increased lung edema, and worsened lung function in a study conducted by Kuba et al. (2006). Gu et al. (2016) observed reduced animal survival and exacerbated lung injury following respiratory syncytial viral infection in mice with genetic deletion of ACE2 gene. Another study in a mice model of *Pseudomonas aeruginosa* lung infection showed an increased pro-inflammatory cytokine and chemokine response, as well as parenchymal inflammation (Sodhi et al., 2019). In the pathogenesis of SARS-CoV infection specifically, Imai et al. (2005) attributed to Ang II upregulation the responsibility for severe lung failure via AT1.

The diminished ACE2 levels may result in: (1) Ang II upregulation, leading to classical RAS axis overactivity; and (2) Ang-(1-7) depletion, attenuating the protective effects of the alternative RAS axis (Figure 2). Ang II is a pro-inflammatory peptide and its upregulation contributes to acute lung injury by promoting endothelial dysfunction and cytokine storm (Rodrigues Prestes et al., 2017). The unbalance between both RAS axes may result in three major disturbances: pulmonary, inflammatory/immune, and hematological. Therefore, as shown in Figure 2, a three-phase disease course was proposed in order to explain COVID-19 pathophysiology and correlate its evolution to RAS activity (Lanza et al., 2020).

*First phase:* ACE2 is expressed in several tissues, including airways epithelium, brain, bone marrow, gastrointestinal (GI) tract, kidney, and heart (Nehme et al., 2015; Li M. Y. et al., 2020). Airways epithelium is the initial site of SARS-CoV-2 infection, from where the virus spreads throughout the body. Therefore, early in the disease course, COVID-19 is remarkably distinguished from other respiratory diseases due to the lymphopenia found in nearly half of the patients at admission (Rodriguez-Morales et al., 2020; Tan et al., 2020). Several possible explanations support this finding, with the four most promising ideas being related to the invasion of different tissues and immune activation. The first one is the invasion of bone marrow. ACE2 is found in hematopoietic stem/progenitor cells (HSPC) and viral induced hypoxia might lead to three main consequences: (1) increased proliferation and migration of HSPC; (2) upregulation of ACE2 and Mas receptor; and (3) shedding of ACE2 ectodomain in HSPC (Joshi et al., 2019). The second hypothesis regards lymphocyte invasion, as this cell expresses ACE2 on its surface and the novel coronavirus is able to invade this cell. The third one considers the deflagration of the hemophagocytic lymphohistiocytosis (HLH), although this mechanism is only enhanced later in disease’s course and might be related to other clinical manifestations. Lastly, studies hypothesized on SARS-CoV-2 interactions with the GI tract, as the function of ACE2 in this system is still unclear and GI symptoms are common in COVID-19 (Li M. Y. et al., 2020; Musa, 2020; Tan et al., 2020).

*Second phase:* the second stage in COVID-19 is the pulmonary involvement. In this regard, Ang-(1-7) depletion might prosecute a key role, given that Ang-(1-7) allegedly reduces lung inflammation, fibrosis, and pulmonary arterial hypertension (Jia, 2016; Santos et al., 2018). In COVID-19 cases with severe manifestations, the disease does not behave as a typical acute respiratory distress syndrome (ARDS) (Gattinoni et al., 2020b). Therefore, the described phenomenon of severe hypoxemia in compliant lungs may be due to a low ventilation-perfusion ratio as a result of lost perfusion regulation and hypoxic vasoconstriction reaction (Gattinoni et al., 2020a; Wang D. et al., 2020). This underlying pathophysiology may be responsible for the high mortality rates in COVID-19 patients that undergo mechanical ventilation (MV), as up to 80% of patients who required MV evolve to death (Richardson et al., 2020). The classical axis exacerbation possibly promotes endothelial dysfunction directly by Ang II effects and indirectly through immune system activation and hypoxia (Hu, 2020; Sardu et al., 2020). In addition, Ang II may act locally in the lungs’ endothelium enhancing ROS production and reducing NO release (Forrester et al., 2018). All these pathophysiological mechanisms might result in vascular leakage and alveolar edema, causing hypoxia and dyspnea (Channappanavar and Perlman, 2017; Leiva-Juarez et al., 2018). In addition to a probable hepatocytes invasion by SARS-CoV-2, an exaggerated inflammatory response might lead to liver injury (Zhang C. et al., 2020). Elevated D-dimer levels, prothrombin time and International Normalized Ratio (INR) and reduced activated partial thromboplastin time (aPTT) are some laboratory findings that might be related to endothelium dysfunction and liver injury, which, in turn, increase the risk for thrombotic and thromboembolic events (Driggin et al., 2020; Huang et al., 2020; Tang et al., 2020; Wu et al., 2020).

There is an important gap between the second and third phases. Immune response and inflammation start right at the beginning of COVID-19, but, as the disease progresses, these mechanisms increase in intensity. SARS-CoV-2 binding to alveolar epithelial cells makes possible for the virus to activate innate and adaptive immune systems, leading to the release of several cytokines, including Interleukin (IL)-6. Previous studies demonstrated that some viral products (as the human immunodeficiency virus TAT protein transactivator) are able to enhance the DNA-binding activity of nuclear factor κB (NF-κB) and nuclear factor IL-6 (NF-IL-6), resulting in increased IL-6 mRNA transcription (Tisoncik et al., 2012; Tanaka et al., 2014). The same mechanism may also occur in COVID-19. In addition, due to the action of pro-inflammatory factors, vascular permeability increases and a large amount of fluid and blood cells get into the alveoli, resulting in dyspnea and respiratory failure (Channappanavar and Perlman, 2017; Leiva-Juarez et al., 2018). The IL-6 is usually synthesized locally on the lesion in the acute stage of inflammation, mediating pleiotropic effects on immune response and hematopoiesis (Tanaka et al., 2014). This cytokine also promotes specific differentiation of naïve CD4+ T cells and is indispensable for T-helper 17 (Th17) differentiation from naïve CD4+ T cells, being related to the disruption of immunological tolerance, auto-immune and chronic inflammatory diseases (Kimura and Kishimoto, 2010; Tanaka et al., 2014). IL-6 moves to the liver through the bloodstream and rapidly induces the release of several acute phase proteins, including C-reactive Protein (CRP) and fibrinogen (Tanaka et al., 2014). On the other hand, IL-6 reduces the production of fibronectin, albumin, and transferrin.

*Third phase:* this last stage is marked by a systemic hyper-inflammatory state named Cytokine Storm Syndrome (CSS) (Mehta et al., 2020). CSS seems to be responsible for worse clinical outcomes and represents an important maker of disease severity (Huang et al., 2020). Indeed, the association between ACE2 downregulation, exacerbation of the ACE/Ang II/AT1 axis and the release of several pro-inflammatory cytokines, including IL-1, IL-6, IL-8, and TNF-α, is well established in literature (Rodrigues Prestes et al., 2017). This finding might be enhanced by the activation of innate and adaptive immune systems triggered by the viral infection itself, which increases the activity of nuclear factors and mRNA transcription of interleukins. In addition, COVID-19 severity is associated with higher levels of IL-6, IL-2R, IL-10, and TNF-α, as well as lower CD4+ and CD8+ T cells (Pedersen and Ho, 2020). Furthermore, SARS-CoV-2 infection could trigger both primary and secondary HLH, an unusual syndrome characterized by fever, splenomegaly, jaundice and the histopathologic finding of hemophagocytosis in bone marrow and other tissues (Fisman, 2000). However, the mechanism by which viruses contribute to HLH development is not fully established. On this wise, previous studies analyzed the association between DNA viruses, the *Herpesviridae* family for instance, and the HLH, mainly because they are potent modulators of the immune response (Brisse et al., 2017). Less frequently, though, cases of HLH arise in RNA virus infections, including Influenza virus and DENV, among others (George, 2014).

Another important issue to be considered is the gut dysbiosis and its potential link to disease progression in COVID-19 (Aktas and Aslim, 2020; Dhar and Mohanty, 2020; Zuo et al., 2020). The detection of SARS-CoV-2 RNA in the stool of some patients and diarrhea in others point to a link between the lung and the intestine. Despite no fecal-oral transmission being reported up to this date, it’s possible that asymptomatic children and adults may shed infectious virus particles in the stool, leading to infection of others (Dhar and Mohanty, 2020). Gut microbiota diversity and the presence of beneficial microorganisms in the gut may play an important role in determining the course of this disease (Aktas and Aslim, 2020; Dhar and Mohanty, 2020; Zuo et al., 2020). Interestingly, gut dysbiosis is present in several risk groups for COVID-19 as well, including the elderly, immune-compromised and diabetic patients (Dhar and Mohanty, 2020). In addition, ACE2 is highly expressed in the luminal surface of the gastrointestinal tract (Nehme et al., 2015), allowing the gastrointestinal tract to be colonized by SARS-CoV-2. This might explain why patients with COVID-19 exhibit gastrointestinal discomfort and diarrhea. The ACE2 loss in the intestine is also related to the hyperactivation of classical RAS axis and the role of gut-lung axis in COVID-19 (Aktas and Aslim, 2020). SARS-CoV-2 infection may lead to degeneration of the gut blood barrier, driving to systemic propagation of bacteria and endotoxins, resulting in a septic shock. In this regard, a pilot study, including 15 patients with COVID-19, found persistent alterations in the fecal microbiome during the time of hospitalization (Zuo et al., 2020). Furthermore, fecal microbiota alterations were associated with fecal levels of SARS-CoV-2 and COVID-19 severity (Zuo et al., 2020). Additional studies are necessary to address the potential role of probiotics in COVID-19.

### Risk Groups

Risk groups are subsets of the population that may probably evolve with the worst prognosis once ill, requiring special attention and more precaution warnings. For COVID-19, some well-established diseases and conditions are considered risk factors: diabetes, hypertension, chronic respiratory diseases, cardiovascular diseases, chronic kidney diseases, and cancer (Nikpouraghdam et al., 2020). Moreover, age and gender differences also seem to outline prognosis variety. Two other non-medical conditions require further investigation. The first one is pregnancy, due to the possibility of vertical transmission of SARS-CoV-2 to the fetus (Simões e Silva and Leal, 2020). The second one is related to the health workers, who are highly exposed to infected people. This exposure may predict a higher viral charge when infection is installed, which could result in higher disease severity. Epidemiological data further support RAS as a protagonist player in COVID-19 pathophysiology. In this regard, we present these risk groups establishing possible connections with RAS unbalanced.

*Age groups:* the most affected population is 50–60 years old (Chen et al., 2020) and epidemiological reports suggest a positive association between severity and aging. Patients with a severe form of disease were significantly older than the rest of the patients enrolled in a study conducted in China (Feng et al., 2020). In addition, the same study showed significantly lower survival rates in patients older than 75 years old, in comparison to younger patients (Feng et al., 2020). This age distribution of COVID-19 prognosis is not only related to the prevalence of preexistent comorbidities, but to the lifespan physiological oscillation of RAS molecules (Chen et al., 2020). In the aging process, there is a decrease in the estrogen/testosterone ratio, which promotes an increase in plasma renin activity, modifying the RAS axes equilibrium (Colafella et al., 2016). Furthermore, children and young adults have higher ACE2 reservoirs than elderly (Zhang P. et al., 2020). Thus, ACE/Ang II/AT1R is upregulated, an essential characteristic for COVID-19 bad evolution. This physiological mechanism is proven by the lower vascular and renal AT2 receptor expression, the raised AT1R expression and the enhanced pressor responsiveness to Ang II in female animal models, as well as the increased sensitivity to Ang II with aging male animal models (Colafella et al., 2016).

*Hypertension and diabetes:* epidemiological studies also showed worse outcome in patients with COVID-19 and associated comorbidities, including arterial hypertension and diabetes mellitus (Gheblawi et al., 2020; Vaduganathan et al., 2020). Indeed, these conditions are closely related to an exaggerated activation of ACE/Ang II/AT1R axis (Rein and Bader, 2017; Nistor et al., 2018; Schiffrin et al., 2020; Zheng et al., 2020). In diabetes, Ang II have been described to exert several deleterious effects, including increase in insulin resistance, endothelial damage and deterioration of renal function (Simões e Silva et al., 2017). Similarly, arterial hypertension courses with an inflammatory state, which includes higher levels of Ang II, chemokines and cytokines, including IL-6 and TNF-α (De Miguel et al., 2015). Therefore, a previous history of RAS imbalance favors the inflammatory state proposed to be responsible for disease severity in COVID-19. On the other hand, once again ACE2 expression might be crucial in prognosis: because several diabetic and/or hypertensive patients take RAS inhibitors to manage the classical axis upregulation.

## RAS Related Medications

The exposed mechanisms about COVID-19 pathophysiology explain the use of RAS-related medications in ongoing clinical trials. In this regard, RAS blocker therapy has been largely discussed on its beneficial or harmful effects. Considering that RAS inhibitors, like angiotensin-converting enzyme inhibitors (ACEi) and angiotensin II type 1 receptor antagonists (ARB), are first-line treatments for hypertension and diabetic nephropathy, some clinical trials aim to investigate whether RAS blockade therapy should be continued or not. Concerns about ACEi therapy are based on the proposition that these medications blunt the conversion of Ang I to Ang II. In addition, ACEi may also increase ACE2 expression and by doing so might favor SARS-CoV-2 binding to ACE2, the receptor for the virus (Fosbol et al., 2020; Perico et al., 2020). In this sense, the Irish CORONACION study (NCT04330300) enrolled 2414 patients with primarily hypertension to evaluate the association between RAS blocker administration and poorer prognosis. Similarly, French ACORES-2 trial (NCT04329195) separated 554 participants in two groups, one continuing RAS blocker therapy and one discontinuing it. Experimental findings, however, show inconclusive data regarding the effect of ACEi upon tissue levels of ACE2 (Bean et al., 2020). The risk of abrupt withdrawal of ACEi and ARB for patients chronically treated with these medications must be taken into account as well. In this regard, a retrospective cohort study using data from Danish national administrative registries concluded that prior use of ACEI/ARBs was not significantly associated with COVID-19 diagnosis among patients with hypertension or with mortality or severe disease among patients diagnosed as having COVID-19 (Fosbol et al., 2020). Therefore, several research groups advocate for treatment continuation in SARS-CoV-2 patients (AlGhatrif et al., 2020; Fosbol et al., 2020).

Despite the concerns about ARB medication by some researchers, other studies propose a potential therapeutic effect of these medications in COVID-19 (Vaduganathan et al., 2020). The general idea is based on the likely enhancement of ACE2 expression following chronic administration of ARB (Rodrigues Prestes et al., 2017). Although the precise mechanisms beyond this upregulation of ACE2 require further characterization, experimental studies on heart, lung, and kidney tissues support this assumption (Rodrigues Prestes et al., 2017). The consequent upregulation of the RAS alternative arm might seem deleterious at first glance, since circulating ACE2 is directly derived from membrane-bound ACE2, minus the anchoring proteins. However, the further protective effects of the ACE2/Ang-(1-7)/MasR axis in the lungs have been considered beneficial in the final balance of RAS blocker therapy, as previously discussed in this article. Blocking the ACE/Ang II/AT1 axis, in this sense, diminishes Ang II lung injury due to its binding to the AT1 receptor and implies upregulation of the alternative arm, as raised ACE2 levels favors the conversion of Ang II into Ang-(1-7) (Rodrigues Prestes et al., 2017). Figure 2 highlights the main effects of Ang-(1-7) binding to the Mas receptor. Additionally, the antagonism of the AT1R by ARAs could enhance the Ang II binding to the AT2 receptor, upregulated under chronic ARA administration, which may also exert protective effects in lung tissue (Vaduganathan et al., 2020). Several clinical trials aim to analyze the clinical outcomes of Losartan administration in patients positive for COVID-19. Two robust American trials aim to analyze the effect of a 7-day administration of Losartan 50 mg in comparison to placebo in patients positive to COVID-19 (NCT04312009, NCT04311177).

A different approach consists on the direct enhancement of the RAS alternative axis by the administration of Ang-(1-7) and Recombinant Human ACE2 (RhACE2). Belgic ATCO Trial will evaluate the results of Ang-(1-7) infusion in comparison to placebo in 60 participants positive for COVID-19 (NCT04332666). The proposed mechanism is based on the tentative of counter-regulating the exacerbation of the RAS classical arm. The administration of RhACE2, on its turn, might have two favorable effects: (1) the upregulation of the ACE2/Ang-(1-7)/MasR axis leading to and its beneficial actions and (2) the functionally neutralization of SARS-CoV-2 in the bloodstream. The second mechanism is possible due to the lacking of the membrane-anchoring domain in RhACE2. Therefore, it would not allow viral entrance into the host cell, but it is rather capable of antagonizing SARS-CoV-2 and preventing its endocytosis (Gheblawi et al., 2020). The binding of the virus to rhACE2 may also stimulate the immune system to counteract SARS-CoV-2 (Gheblawi et al., 2020). Although being promising therapeutic alternatives to COVID-19, treatment with these peptides face some major challenges, including the short half-life *in vivo*, low stability, high manufacturing cost and rapid degradation in the gastrointestinal tract when administered orally, meaning the need of a continuous intravenous infusion (Shenoy et al., 2015). In this sense, the one trial proposing RhACE2 administration to treat COVID-19 still lacks the Center of Drug Evaluation approval (NCT04287686). Further pharmacological investigation on these peptides could represent a brand new therapeutic perspective for COVID-19, as well as for other diseases related to RAS imbalance.

## Concluding Remarks

Coronavirus Disease 2019 pathogenesis and pathophysiology are far from being fully elucidated. The first studies considered different stages of the diseases separately and without well-established mechanisms. In this context, we do believe that RAS imbalance may exert an important role in COVID-19. The shift of RAS equilibrium toward the classical axis, ACE/Ang II/AT1R, in parallel with downregulation of the alternative axis, ACE2/Ang-(1-7)/MasR, may contribute to the plethora of clinical manifestations of COVID-19 and its severity. In this regard, we defend that the novel therapeutic and preventive strategies take into account the importance of restoring RAS equilibrium.

## Author Contributions

LC and LP made the literature revision and selection of main manuscripts and wrote the first draft of the review. VP, TM, and VR defined the topics of this review and helped in writing the first draft. TM and LC made the figures. AS and KL conceptualized the study, made general supervision, and revised the manuscript. AS submitted the final version of the manuscript, which is approved by all authors.

## Conflict of Interest

The authors declare that the research was conducted in the absence of any commercial or financial relationships that could be construed as a potential conflict of interest.
